# Factors influencing obstetricians’ intentions to facilitate normal physiological birth: a qualitative study

**DOI:** 10.1186/s12884-026-09247-2

**Published:** 2026-06-09

**Authors:** Holly Meyer, Nigel Lee, Kendall George, Lauren Kearney

**Affiliations:** 1 School of Nursing, Midwifery and Social Work, University of Queensland, Level 3, Chamberlain Building, St Lucia, Brisbane, QLD 4072 Australia; 2 School of Health, University of the Sunshine Coast, Sippy Downs, Australia; 3 Women’s and Newborn Services, Royal Brisbane and Women’s Hospital, Metro North Health, Herston, Australia; 4 Women’s and Newborn Services, Townsville Hospital and Health Service, Townsville, Australia

**Keywords:** Normal birth, Obstetrics, Qualitative Research, Theory of Planned Behaviour, Influences, Collaboration, Education

## Abstract

**Introduction:**

Spontaneous vaginal birth is the preferred mode of birth for most women, however, increasing intervention has decreased rates, especially for nulliparous women. Obstetricians are identified as key stakeholders in intrapartum care, however very few studies have focused on the role obstetricians’ intentions, attitudes and behaviours may have on mode of physiological birth. The purpose of this study was to explore factors influencing the intentions of obstetricians to facilitate normal physiological birth.

**Methods:**

This qualitative study was underpinned by the Theory of Planned Behaviour. All participants were obstetricians, registered and practising within Australia, and had experience providing care to birthing women who met the ‘selected primipara’ criteria. Semi structured interviews were conducted with eight obstetricians representing various models of care across most States and Territories of Australia. Interviews were analysed using thematic analysis.

**Results:**

Two key themes were identified from the data, with three sub-themes nested within each. Theme 1 identified the ‘Knowledge and beliefs’ influencing participants intentions to facilitate normal physiological birth, including ‘Definitions and nomenclature’, ‘Knowledge acquisition’, and ‘Communication and litigation’. Theme 2 identified ‘External influences’ impacting participants intentions to facilitate normal physiological birth and included ‘Colleagues’, ‘Structures – policy, resources and models of care’, and ‘Women and the Community’.

**Conclusions:**

While obstetricians aimed to support the enabling of normal physiological birth, their intentions were influenced by many factors. Facilitating interdisciplinary interactions and education, embedding woman-centred care and promoting midwives’ autonomy and full scope of practice would further alleviate known barriers.

**Supplementary Information:**

The online version contains supplementary material available at 10.1186/s12884-026-09247-2.

## Background

Interventions during labour and birth have increased in recent decades, with the aim to reduce maternal and neonatal morbidity and mortality [1–3]. While there is no argument that access and availability to safe birth interventions are needed, international debate continues over “too much too soon” or “too little too late” [3]. Documented increases in birth interventions have not afforded substantial improvements in recent years in overall maternal and neonatal morbidity [4–8]. International guidelines [4, 9] acknowledge the importance of normal physiological birth to improve outcomes for women and their babies, with practice to promote normal physiological birth as a key focus within their recommendations. Various definitions for ‘normal physiological birth’ exist amongst the literature [10–13], however, for this study has been defined as spontaneous onset of labour, at term, without augmentation or pharmaceutical pain relief, and concluding with the unassisted vaginal birth of the neonate and placenta.

Previous studies support the premise that while low-risk nulliparous women are more likely to be exposed to iatrogenic interventions they are also more likely to benefit from the promotion of normal physiological birth [14, 15]. Hence obstetricians are central to the facilitation or hindrance of normal physiological birth, as they are frequently the final decision-makers for birth interventions, such as induction of labour, instrumental birth and casesarean section [16]. Past research highlights how the implementation of clinical practices such as: revised criteria for labour dystocia; midwifery continuity of care; and, increasing mobility during labour and birth, could improve normal physiological birth rates [3, 17, 18]. However, few studies have focused on the role that obstetricians’ intentions, attitudes and behaviours may have on normal physiological birth. Applying a theoretical framework to explore factors that influence obstetricians’ intentions may provide insights previously not explored.

A central tenet of the Theory of Planned Behaviour (TPB) is that individuals who possess both the intention and perceived control to perform a behaviour are likely to enact it. The TPB seeks to investigate the many causal factors that may influence this [19]. While the TPB has previously been applied to understand behaviour and decision-making in various healthcare settings [18, 20, 21], its use within maternity care, in particular obstetric practice, remains limited. Although literature exists describing obstetricians’ attitudes, beliefs, and risk orientations in relation to labour interventions [22, 23], there remains a notable gap in studies employing strong theoretical underpinnings such as the TPB to examine the underlying mechanisms shaping these practices, especially regarding normal physiological birth. Utilising the TPB framework, this study aims to investigate the attitudes and beliefs, subjective norms, and perceived behavioural controls influencing obstetricians’ intentions to facilitate normal physiological birth.

## Methods

### Study design

A qualitative approach, underpinned by the TPB [24] was employed.

Following predetermined inclusion and exclusion criteria a purposive sampling strategy was employed [25]. Obstetricians were invited to participate if they were registered through Australian Health Practitioner Regulation Agency and had recent clinical experience supporting women who met the *‘selected primipara’* criteria: mothers aged 20–24 years old, giving birth for the first time at > 20 weeks’ gestation, with a singleton pregnancy, in vertex presentation, birthing at term (37–42 completed weeks gestation). Obstetricians practising in public and/or private sectors (including Diplomats in General Practice) were invited.

Recruitment material was shared via social media platforms as well as word of mouth. Obstetricians meeting the study criteria were invited to participate. Twenty-four obstetricians indicated interest in participating at recruitment. Scheduling challenges, including non-responses and purposive refinement guided by demographic data, reduced the final sample to eight participants. Those responding positively were provided with further information. Upon receipt of written consent to participate, an interview was arranged at a mutual convenient time.

### Data collection

Individual interviews between the participants and the first author (HM) were conducted via video conferencing application, lasting between 34 min and 1 h 6 min in duration, between July 2022 – February 2023. An interview guide employing the TPB framework, was utilised to facilitate discussion and ensure the key objectives of the interview were met [19]. The interview guide was pilot tested, resulting in the condensation of some questions to ensure suitability and relevance for the obstetrician participant group. Questions explored obstetricians’ knowledge and beliefs (e.g. *Can you tell me your understanding of normal physiological birth?*), social influences (e.g. *Are there individuals/groups close to you that support or inhibit you to facilitate normal physiological birth?*), and factors perceived to be out of their control regarding normal physiological birth (e.g. *What factors/circumstances do you think makes it easier/difficult to facilitate normal physiological birth?*). Audio recordings were obtained from the video-conference application and transcribed by the interviewer. Contemporaneous field notes were taken during the interviews, to facilitate analysis and interpretation of individual data [26]. The principles of reflexive thematic analysis informed the final sample size, by prioritising the need to achieve richness and complexity of data sufficient to generate meaningful patterns across the dataset [27].

### Data analysis

Braun and Clarke’s [28] reflexive thematic analysis was used to thematically code the data. The framework presents a systematic and rigorous, yet flexible, approach to coding and theme development including data familiarisation, generation of codes and potential themes, theme definition, examination of final themes, and report generation. Consistent with reflexive thematic analysis, an inductive, deductive approach was employed by the first author (HM) using NVivo (Version 12 Plus), with the TPB framework, Knowledge and beliefs, Social norms, and Perceived behavioural control, underpinning the initial deductive analysis for this study [29]. The first author is an early career researcher conducting a PhD with wide-ranging midwifery practice experience. Regular discussions with the research team (all experienced with normal physiological birth) of the findings, codes and themes enhanced rigour. These discussions ensured a more robust and nuanced understanding of the data through challenging of assumptions and exploring other interpretations.

## Results

Eight obstetricians across public, private and GP practice models of obstetric care were interviewed. Participants were from most states and territories of Australia, with practice experience ranging between 5 and 40 years (Table 1). Two overarching themes were constructed. *Theme 1: Knowledge and beliefs*, including sub-themes *Definitions and nomenclature; Knowledge acquisition;* and *Communication and litigation. Theme 2: External influences* including sub-themes *Colleagues; Structures – policy*,* resources*,* and models of care;* and *Women and community.* Each sub-theme is illustrated by quotations that are anonymised using participant pseudonyms.

**Table 1:** Participant demographics - obstetricians

Pseudonym	Age (years)	Sex	Model of Care	State	How long as an obstetrician (years)
P1_OB	35-45yrs	Female	Public Hospital	Queensland	0-5yrs
P2_OB	55-65yrs	Female	Public Hospital	Queensland	30-40yrs
P3_OB	25-35yrs	Female	GP Obstetrician/Public Hospital	New South Wales	0-5yrs
P4_OB	35-45yrs	Female	GP Obstetrician/Public Hospital	Northern Territory	10-15yrs
P5_OB	35-45yrs	Female	Private & Public Hospital	South Australia	0-5yrs
P6_OB	35-45yrs	Female	Private & Public Hospital	New South Wales	5-10yrs
P7_OB	45-55yrs	Female	Public Hospital	Victoria	20-30yrs
P8_OB	45-55yrs	Male	Private & Public Hospital	Queensland	15-20yrs

### Knowledge and beliefs

#### Definitions and nomenclature

Participants understood normal physiological birth as commencing spontaneously, progressing without complication, and concluding in the birth of the baby. They also included the option of pain relief and medications to actively manage the birth of the placenta, within this definition. Participants believed these interventions were necessary to mitigate perceived risks associated with normal physiological birth.

*“… if we use certain medications for helping with the placenta that minimises blood loss*,* and to me that is part of a spontaneous vaginal birth”. – P7OB*.

*“where labour is left to spontaneously start there’s naturally going to be an increased stillbirth rate with a longer gestation …*,* if you’re talking physiological third stage*,* then an increased postpartum haemorrhage rate”. – P5OB*.

Participants generally recognised the importance of facilitating normal physiological birth where reasonable, highlighting benefits such as improved postnatal recovery and breastfeeding compared to caesarean section. Participants recognised the advantages of normal physiological birth for women however, this was caveated with careful monitoring for variations from a normal parameter. There was a tendency for participants to bring conversations back to concerns about risk when discussing normal physiological birth.

*“The pros are you probably increase breastfeeding rates… probably a shorter length of stay in hospital” – P5OB*.

*“If you talk about things like perinatal mortality*,* then caesarean section is probably the safest. … babies generally don’t die from caesarean sections. Whereas intrapartum*,* it can be more unpredictable. That’s the reality… the intrinsic risk of vaginal birth … But now we can bypass that risk”. – P6OB*.

Other participants expressed reservations about using terms like “normal” and “physiological” to describe birth in its simplest form, suggesting that for some women, opting for a planned caesarean section was considered normal, and they should not be made to feel otherwise in their birth choices. Similarly, the concept of planned caesarean section as a means of avoiding the unpredictable nature of normal physiological birth and to improve perinatal morbidity and mortality rates was also raised.

*“But in private practice*,* it’s a little bit different because lots of women come and see us because they don’t want a vaginal birth. So I suppose to them a normal birth is a caesarean section. So I think it’s about working out what it is for the patient. That’s the most important thing” – P5OB*.

*“So*,* I have a huge problem with ‘normal’*,* and ‘physiological’ because it seems to then kind of create a dichotomy of if you didn’t have a normal birth*,* then it must have been an abnormal birth.” – P7OB*.

#### Knowledge acquisition

Participants believed that their understanding of normal physiological birth came from their education, training, and practical experiences. They described their foundational training as focused on mechanisms of birth rather than providing a comprehensive understanding of the woman’s experience. Participants explained that they were trained to be risk averse, with a primary focus on complex and obstetric emergency management. This focus was often reinforced by clinical experiences, further shaping their perceptions of normal physiological birth as something to approach with caution.

*“I learned about physiological birth*,* but it was a mechanism rather than an event that defines a woman’s life.” – P8OB*.

*“We’ve been trained to be risk averse… trained in managing emergencies and all the risks that go with labouring. When you are only doing the risk stuff and not doing any of the normal stuff*,* it’s very difficult for those to align in the way that you manage birth.” – P1OB*.

While participants initial training developed basic understanding, they believed that they gained deeper insight into normal physiological birth through their interactions with their midwifery colleagues. Participants valued working closely with midwives, particularly in small birthing units, as it allowed them to acquire insights and skills to support and promote normal physiological birth.

*“The basics were from my training… But it probably hasn’t had the hugest influence on what I do now… I work in small a small unit very closely with midwives. I’ve learned a lot from them*,* too… I probably draw a lot of my practice from how our midwifery team does things”. – P3OB*.

*“we’re not experts in normal birth. I learn things from midwives all the time” – P8OB*.

#### Communication and litigation

Effective communication was consistently highlighted as essential for facilitating normal physiological birth, however, in practice it was often centred on translating risk rather involving women in shared decision-making. Participants emphasised the importance of informing women about risks, however also acknowledging that the fear of litigation influenced how certain information was framed, subtly shifting communication from shared decision-making to liability mitigation.

*“And all we want is that you understand that risk. And with statistics*,* … it depends on how you put it. If I wanted to sway you into accepting what I’m saying*,* I would say it’s three times the risk”. – P7OB*.

*“The things that inhibit the ability of that is probably you know*,* the medical indemnity kind of stuff that happens… it’s always in the back of your mind. We have 100% litigation rate… over the course of our career”. – P5OB*.

Selecting the woman’s ‘suitability’ for normal physiological birth was identified as a necessary strategy to avoid unwanted risks and in turn reduce exposure to potential litigation. More experienced participants believed that the fear of litigation diminished with experience and confidence in practice, though they acknowledged it continued to influence them to intervene more quickly. This approach conflicted with their stated commitment to involving women in decision-making, as defining ‘suitability’ positioned professional risk assessment above women’s preferences and autonomy.

*“In trying to support women to have a normal physiological birth. It’s about selecting the right women… I probably select out the right people to labour. You know*,* the short Tubby women who have big babies with big heads …*,* I’m not going to put them through labour. That’s reality. And I will say that to them*,* and sometimes they will try. But they at least have been fully informed of what the possible outcomes are”. – P6OB*.

### External influences

#### Colleagues

Colleagues were perceived to have significant influence on participants intentions to facilitate normal physiological birth. The attitudes held by obstetric colleagues regarding normal physiological birth were perceived as either a barrier or enabler and could impact on their ability to promote normal physiological birth within their workplace.

*“I definitely have colleagues who actively discourage women to have any kind of vaginal birth”. – P6OB*.

*“I think among some obstetricians*,* there’s an anti-promotion of normal birth*,* kind of sentiment and that sometimes comes through*,* and some people are more risk averse than others”. – P8OB*.

Fragmented care within large public hospitals was particularly impactful due to the involvement of numerous clinicians in women’s care. Information shared with women, as well as actions taken by other obstetric colleagues varied, and were viewed as beyond their control, though still influenced the care provided to women. Consultant obstetricians explained that moderating the actions of junior obstetricians and midwives was sometimes required to reduce interference. Avoiding continuous fetal monitoring and unnecessary interventions were key strategies used. Others identified that input from both obstetric and midwifery colleagues within the birth suite led participants to reconsider their own clinical decisions, affecting not only their actions but also their perceived autonomy.

*“It’s those external influences. It could be from a more senior midwife. It could be from a consultant or another registrar… people who maybe walking through the birth suite … I probably am a bit influenced”. – P1OB*.

*“I suppose the other way that I probably facilitate it now as a consultant is putting the brakes on my registrars”. – P1OB*.

Participants described a divisive culture between midwives and obstetricians. This perceived barrier at times hindered efforts for teamwork, with some attributing it to midwifery colleagues and others believed it was perpetuated by leadership and hierarchy. Horizontal violence experienced within the workplace negatively affected the level of commitment participants had to promoting normal physiological birth. The divide between the two disciplines was also identified by participants to extend beyond the workplace and was evident at a broader cultural level within the community.

*“It was not so much a team working together*,* it was more an us verse them. But that all comes from the top”. – P1OB*.

*“I had a midwife stand at the door and say ‘you can’t come in*,* we don’t need you’… And I found that really upsetting… I suppose there’s always that divide right like*,* doctor*,* midwife” – P3OB*.

*“I can influence the people who are within my sphere of influence*,* but that ‘us’ and ‘them’ that seems to exist on a broader scale is out of my control*,* and that is unhelpful”. – P7OB*.

Participants working in private hospital settings stated that midwives tended to involve doctors earlier, contributing to a workplace culture that lacked confidence in normal physiological birth. Establishing respectful interprofessional relationships, especially with midwives, was deemed as essential for successful facilitation of normal physiological birth. Supporting midwives to work to their full scope of practice was identified as strategy to promote normal physiological birth.

*“The midwifery teams working across both public and private are vastly different. I get calls*,* much earlier in the private hospital to intervene… I think it’s probably just the expectation*,* the culture. … they’re expecting it*,* and the midwives have set that dynamic up*,* throughout their labour care”. – P5OB*.

*“Why aren’t midwives paid to work to their full scope? Why aren’t they primary care providers for most women who don’t need intervention? And obstetricians getting involved when they’re needed?” – P8OB*.

#### Structures – policy, resources & models of care

Workplace structures, such as policies and resource allocation were considered to be obstacles to promoting normal physiological birth. Participants identified that hospital policies were overly restrictive, hindering their ability to facilitate normal physiological birth.

*“I think protocols and procedures*,* that’s the biggest thing. Any birth in hospital is going to be driven by… exclusion criteria for this and that*,* that we even in a private hospital have to follow… So that I think is tricky for women. We are bound by those as well and that does limit my practice”. – P5OB*.

The level of resourcing in their place of practice was another constraint on participants intention to facilitate normal physiological birth. Participants working in private hospitals experienced restriction due to limited theatre staffing during certain times, such as after hours. Similarly, in the rural setting, a lack of access to qualified staff and services impacted their decision-making and hindered efforts to promote normal physiological birth for fear of reduced safety.

*“In private [hospitals]*,* I think they are more proactive and definitely more interventional… probably because of the lack of support after hours. Whereas in the public [hospitals]*,* there’s always staff around*,* there’s theatres on 24/7”. – P6OB*.

*“I think I tend to probably call it a bit earlier. And part of that is just knowing that we don’t have access to everything that you do in a bigger hospital. So*,* if I’m going to make that decision*,* I’m better off to be making it at 6pm than 3am*,* when you’ve now got a severely obstructed labour”. – P3OB*.

Continuity of care was identified, by most, to be a supportive mechanism for facilitating normal physiological birth. Participants working in private hospitals believed that the ability to maintain continuity with women throughout the childbirth continuum had advantages, such as fostering trust and facilitating information sharing. However, those working in public hospital settings felt disadvantaged by the lack of continuity afforded to them. Continuity of care in private obstetrics was recognised to only be supportive of normal physiological birth if the obstetrician’s attitudes aligned with this approach. Midwifery group practice models were recognised to have a positive impact through enabling midwives to facilitate normal physiological birth.

*“We have a discussion with our patients*,* that’s a benefit*,* I guess*,* of having continuity of care is that you can start that discussion at 10 weeks and continue the discussion right up until a woman’s in labour… but you also have to be more careful not to overstep that*,* they’ll do whatever you say*,* because you build this trust with them”. – P5OB*.

*“I can see continuity of care is important. And certainly*,* private obstetricians is a continuity of care model*,* but it’s not a promotion of normal birth model”. – P8OB*.

*“So having that one-on-one care for women with midwifery group practice*,* I think has been really helpful in facilitating physiological birth”. – P1OB*.

#### Women and the community

Participants believed that birthing women had significant influence on obstetricians’ ability to facilitate normal physiological birth. Women’s mindsets and birth preferences, often shaped by external influences such as culture, friends, and family, were identified as key factors. Broader community attitudes amplified through social media were also recognised as influential to women’s decision-making regarding birth. Women’s preferences for or against normal physiological birth, often observed on a spectrum of two extremes, was considered to be out of the participant’s control, impacting their ability to facilitate normal physiological birth regardless of intention.

*“It swings back and forth as to what people*,* community*,* birthing women want*,* really. And that really drives our practice.”. – P5OB*.

*“It’s tricky to have those complex discussions with patients about where their birth expectations are coming from*,* because you’re not in their Instagram feed 10 times a day. Lots of my private women*,* it’s watching [celebrity personality] with an elective caesarean section*,* her body back like two weeks later. For some*,* that’s the standard that they’re trying to get to. And for other people*,* it’s no drugs waterbirth*,* all of that”. – P5OB*.

*“Social media has a lot to answer for”. – P2OB*.

In contrast to participants working in metropolitan areas, those practising in rural and remote settings noted that women were often motivated to pursue a normal physiological birth for practical reasons, such as faster recovery and a prompt return to home and normal daily activities.

*“We have a lot of farmer’s wives. People that live on a rural station*,* and they’re always really keen to have a normal birth*,* because then they can just go back to station work”. – P4OB*.

Participants conveyed being challenged to varying degrees, by women’s birth preferences, particularly when they were not aligned with recommended care. While participants were mostly willing to support women’s choices, concerns were expressed that women’s expectations were unrealistic. Participants with experience working in private practice were more comfortable supporting women choosing a more actively managed birth, including caesarean section for social reasons if requested.

*“I’ve spent a lot of my time looking after women who have challenged the system and I find that quite satisfying in lots of ways*,* because those women will challenge my own beliefs*,* knowledge*,* etc.”. – P2OB*.

*“…more women are working so they need to organise their lives. Fair enough. Why shouldn’t they be organising their lives around other children*,* around family members and you know*,* wanting to have an induction on a particular day or book a caesarean on a particular day. I can’t see that it is problematic by itself. But of course*,* it’s problematic for facilitating a normal physiological birth”. – P7OB*.

### Discussion

Our findings highlight that obstetricians’ intentions were ultimately to support and promote the facilitation of normal physiological birth, however their decision-making and subsequent practices were heavily influenced by workplace structures and cultural norms.

Variability in obstetrician’s understanding of normal physiological birth was demonstrated, with definitions often including intervention aiming to mitigate risk. A significant influence on knowledge and attitude was their foundational training, primarily focused on the mechanics of birth and risk management and later shaped by experiences in the clinical area. Participants recognised that this training conditioned them to be risk averse through curricula that focused on birth mechanics, obstetric emergencies, and strategies for managing risk. This finding is not surprising given that the Australian obstetric prevocational curriculum centres on knowledge surrounding surveillance and complex obstetric skills [30]. A Canadian study by Klein, Liston [31] suggests that obstetricians’ risk-bias and views towards birth are developed during training, with younger obstetricians often being more risk averse. Contrary to the findings of previous studies [16, 32], senior obstetricians in this study were more likely to be supportive of normal physiological birth and employed strategies to reduce interventions instigated by less experienced colleagues.

This study indicated variation in participants attitudes, beliefs, and subsequent practices surrounding normal physiological birth. While consensus was that normal physiological birth is beneficial for most women, given the right circumstances, there was obvious tension regarding how best to approach this area of practice. A clear risk bias was evident, with some participants expressing scepticism of normal physiological birth and demonstrated a preference for a more managed and controlled approach to birth. These attitudes and beliefs are consistent with previous research which highlight that clinicians’ perceptions of risk influence their clinical practice and decision-making, with personal beliefs being the largest contributing factor [16, 33]. The TPB [34] supports this, and asserts that a person’s attitudes towards a behaviour significantly influence their intention to perform the behaviour. Given the influence of knowledge acquisition on participants attitudes towards normal physiological birth, and subsequent behaviour, it could be assumed that foundational obstetric training requires reform.

Social dynamics in the workplace were identified to have considerable influence on participants’ intentions to facilitate normal physiological birth, with colleagues seen to have both positive and negative effects. Interventions initiated by other clinicians, such as induction of labour and fetal surveillance, reduced participants sense of autonomy and self-efficacy and influenced the care provided. This posed as challenging within the workplace especially when attitudes and beliefs towards normal physiological birth were in opposition. This finding aligns with previous research, highlighting how care is often impacted by the attitudes and beliefs held by other clinicians, particularly where these were misaligned [18, 35]. These findings highlight the need to prioritise strategies that address these workplace cultural barriers to ensure alignment amongst the maternity care workforce to better support the facilitation of normal physiological birth.

Findings from this study suggest that entrenched professional hierarchies within the maternity care system play a significant role in shaping obstetricians’ intentions and the culture of the workplace. Despite the presence of national frameworks promoting interprofessional collaboration, including the Australian College of Midwives *National Midwifery Guidelines for Consultation and Referral* [36], and the *National Safety and Quality Health Service Standards* [37], obstetricians in this study were often positioned as authoritative leaders and ultimate decision-makers in birth care. This hierarchical positioning is valued and reinforced within institutional structures and by obstetric professional organisations that advocate for obstetricians to hold primary administrative responsibility across all maternity services [38]. While some participants affirmed this authoritative role, others identified a need to increase the scope and strengthen midwifery leadership within maternity care systems, with obstetricians adopting a collaborative and supportive function where appropriate.

Despite these professional hierarchies and recognised differences in birth philosophies, participants acknowledged and respected the expertise midwives held in facilitating normal physiological birth. Many participants actively sought midwives’ guidance and advice to better their understanding and support normal physiological birth, highlighting how the interprofessional relationship was integral to the successful facilitation of normal physiological birth. This finding is contrary to previous research, which have suggested tensions between midwives and obstetricians, often grounded in differences in philosophies, perceptions of autonomy and decision-making authority [22, 33, 39]. Findings from this study highlights the opportunity to strengthen relationships and shared knowledge between the two disciplines. Existing evidence suggests that shared learning between midwives and obstetricians can improve confidence and support around birth and cultivates long-term collaborative practice [40–42]. While much of this research has occurred in contexts outside the Australian maternity system, an Australian study also supports these findings [43]. Educational strategy has potential to provide obstetricians with greater confidence to promote normal physiological birth, alleviate risk bias and develop a stronger more connected maternity workforce.

Litigation was an influential factor on participants practice, particularly in their approaches to information sharing and willingness for use of interventions. The study found that participants’ intentions to facilitate normal physiological birth could be influenced by fear and the perceived threat of litigation. This was compounded by system-level pressures, including organisational priorities, limited resourcing and fragmented care. This finding is not surprising and is consistent with previous research which highlights the impact of litigation concerns and hospital system priorities on clinical practice, often resulting in increases in intervention [16, 32, 33, 44]. Bisits [45] similarly argues that an overabundance of information, such as research and clinical guidelines, creates a culture of fear, in which attempts to satisfy medico-legal risk-communication obligations unintentionally contribute to overtreatment. Our findings extend this understanding, showing that litigation not only impacts care provided but also influences the way information is communicated with birthing women. Fear of litigation contributed to a tendency for participants to offer birth options that were more predictable and controlled, believing that this minimised perinatal morbidity and mortality rates and in turn litigation risk.

Participants highlighted effective communication with women as a key strategy to facilitating normal physiological birth. However, the nature of communication often prioritised risks, with information selectively shared to direct women toward birth options that aligned with system priorities, participants’ preferences and risk tolerance. Some participants deliberately censored information about birth choices believing this would not only limit risk to women but also themselves. This behaviour reflects what Collins, Burns [46] describe as ‘grooming’ or ‘cultivating compliance’ whereby care providers seek compliance to align with health system needs through frequent discussions prioritising risk management strategies above women’s own preferences. Such practice raises concerns regarding informed consent and the autonomy of the birthing women, due to the imbalance of risk management and informed, woman-centred care. While participants intentions were to support normal physiological birth, these findings highlight the power imbalance between obstetricians and birthing women, also acknowledged in previous research [47, 48]. Further, such practices demonstrate a significant departure from a woman-centred care approach promoted by the World Health Organization [4] and highlight how broader structural and contextual mechanisms impact on decision-making [16, 33].

The sound methodological framework of the TPB [24] that guided data collection and analysis in this study adds rigour and confidence to the findings. The TPB provided a rigorous structure to explore individual intentions, however broader social structures impacting intentions were not theorised in depth. The sample included participants from both public and private obstetric models of care and from most states and territories of Australia. While the sample includes diversity in workplace settings and experiences, it was overly represented by female obstetricians, with only 1 male obstetrician participating in the study. This ratio could be explained by the current workforce that has an increasing female population [49]. As participation was self-selected, the sample may reflect those with particular interest in the topic, which may limit transferability of the findings. A further limitation is that the research team comprised only midwives, which may have influenced interpretation of the findings. Due to the qualitative nature of this study the findings are not intended to be generalised across the broader population. However, the rich qualitative data gathered adds strength to understanding underlying attitudes influencing intentions for practice.

### Conclusion

Many factors influenced obstetricians’ intention to support normal physiological birth. This study highlights variation in obstetricians’ understanding, attitudes, and approaches to normal physiological birth, shaped by factors such as risk-averse training and clinical experiences, workplace culture, and systemic structure influences. Fear of litigation and health system preferences were barriers that influenced practice, particularly regarding communication strategies, and raised concerns around imbalances between informed consent and women’s autonomy. The factors perceived to be outside of their control were the attitudes and behaviours of others, including birthing women and obstetric colleagues, however these were also influential in their ability to facilitate normal physiological birth. However, collaborating with and acknowledging and valuing midwives as the experts in normal physiological birth was considered supportive and is a key area to strengthen through shared learning. This approach has potential to strengthen interprofessional relationships, reduce risk bias, and support facilitation of normal physiological birth. Further, peer review and reflective practice, consistent with standards within the midwifery profession, may afford similar benefits in obstetrics. Future research should build on these findings by exploring strategies to reform foundational training, address workplace cultural barriers, and promote collaborative care that centres women as leaders in their childbirth experience.

## Supplementary Information

Supplementary Material 1

## Data Availability

The datasets generated and analysed during the current study are not publicly available due to confidentiality restrictions required but are available from the corresponding author (HM) on reasonable request for projects with ethical approval.

## Abbreviations

TPB: Theory of Planned Behaviour
