# Supplementary material for: Factors influencing obstetricians’ intentions to facilitate normal physiological birth: a qualitative study
Source: BMC Pregnancy Childbirth. 2026 Jun 9;26:897. doi: 10.1186/s12884-026-09247-2 (PMC13474865; doi:10.1186/s12884-026-09247-2)
Supplement: Supplementary file 1 [file 12884_2026_9247_MOESM1_ESM.docx]

*Interview Guide – Obstetricians*

***Knowledge***

- Can you tell me your understanding of normal physiological birth?
- Where did you draw that knowledge from?
- How do you facilitate normal physiological birth?
- Has any particular information/practice influenced how you facilitate normal physiological birth?

***Behavioural beliefs***

- What do you believe are the advantages/benefits of normal physiological birth?
- What do you believe are the disadvantages of normal physiological birth?
- What do you believe are the risks of normal physiological birth?
- How important do you think it is to facilitate normal physiological birth? Why?
- Do you think most nulliparous women can achieve a normal physiological birth? Why/Why not?

***Subjective norms***

- Are there individuals/groups close to you that **support or encourage** you to facilitate normal physiological birth in the workplace?
- Are there individuals/groups close to you that **inhibit or discourage** you to facilitate normal physiological birth in the workplace?
- Are there social/societal structures that **support or encourage** you to facilitate normal physiological birth in the workplace? Social/causal structures = features of the world that precede and succeed our individual lives, though can be changed over time. Eg. institutional structures, law, ideology, culture

Expectations from others and motivation to comply

- Are there social structures that **inhibit or discourage** you to facilitate normal physiological birth in the workplace?
- Who/what has the most influence on your decisions?
  - How does this impact your intention to facilitate normal physiological birth?
- Do you believe you have agency/ capability to act independently/make independent decisions, to facilitate normal physiological birth?
- Do you believe that you have the capacity/can facilitate normal physiological birth?
  - How do you think that impacts on your intention/aim/goal to facilitate normal physiological birth?

***Perceived behavioural control***

- What factors/circumstances do you think makes it easier/motivates to facilitate normal physiological birth?
- What factors/circumstances do you think make it difficult/prevents to facilitate normal physiological birth?
- Are there things that you feel are out of your control that impact your intention/aim/goal to facilitate normal physiological birth? What?
- What, if anything, would you like to change?
- Do you have any further comments to make?
